# Vascular and Urinary Tract Anatomic Variants Relevant to Para-Aortic Lymphadenectomy in Women with Gynecological Cancers

**DOI:** 10.3390/cancers15204959

**Published:** 2023-10-12

**Authors:** Nina Kovačević, Marko Hočevar, Gregor Vivod, Sebastjan Merlo

**Affiliations:** 1 Department of Gynecological Oncology, Institute of Oncology Ljubljana, 1000 Ljubljana, Slovenia; nkovacevic@onko-i.si (N.K.); mhocevar@onko-i.si (M.H.); gvivod@onko-i.si (G.V.); 2 Faculty of Medicine, University of Ljubljana, Zaloška cesta 2, 1000 Ljubljana, Slovenia; 3 Faculty of Health Care Angela Boškin, Spodnji Plavž 3, 4270 Jesenice, Slovenia

**Keywords:** para-aortic lymphadenectomy, gynecologic malignancy, ureter, renal artery, renal vein, inferior vena cava, aorta, anatomy, urinary tract

## Abstract

**Simple Summary:**

Para-aortic lymphadenectomy is an essential part of gynecologic oncologic surgical treatment. The surgeon should be aware of the complex usual anatomy and its common variants. Vascular and urinary tract anatomic variants are common and may be found in one out of five patients during para-aortic lymphadenectomy, so the surgeon should always consider them. The most common vascular variant is the accessory renal artery, which is usually not detected on preoperative imaging. An abdominal CT should be interpreted jointly by a radiologist and a surgical gynecologist whenever possible. For optimal intraoperative management, it is strongly recommended that precise dissection be performed to facilitate exposure and provide valuable insight for potential vascular repair.

**Abstract:**

Background: Para-aortic lymphadenectomy is an essential part of gynecologic oncologic surgical treatment. The surgeon should be aware of the complex usual anatomy and its common variants. Methods: Between January 2021 and May 2023, 58 women underwent para-aortic lymphadenectomy for gynecologic malignancies. Results: Vascular and urinary tract anatomic variants were retrospectively reviewed from the prospective institutional database and results were compared with preoperative contrast-enhanced abdominal CT. Of these 58 women, 47 women had no vascular or urinary tract variants. One woman had a double inferior vena cava, two patients were found to have a retro-aortic left renal vein, four had accessory renal arteries, two had a double left ureter, one had a ptotic kidney in the iliac fossa, and one patient had bilateral kidney malrotation. Anatomic variants in the preoperative CT were described by a radiologist in only two patients, and additional vascular and urinary tract variants were found incidentally at the time of surgery. Conclusions: Acknowledgment of vascular and urinary tract variants is helpful for the surgeon to establish an appropriate surgical plan and to avoid iatrogenic surgical trauma.

## 1. Introduction

Gynecologic cancers continue to be a concern, primarily because of their high incidence rates and resulting cancer-related mortality. Our goal should be early detection of precancerous lesions or early-stage cancer. Currently, there are only prevention programs for cervical cancer, and there are no screening programs for early detection of endometrial or ovarian cancer. Therefore, when diagnosing these two types of gynecologic cancer, it is critical to perform a thorough staging procedures [1].

Para-aortic lymphadenectomy is an integral part of radical gynecologic oncologic surgery for a variety of gynecologic malignancies. Para-aortic lymphadenectomy is performed in indicated cases as part of complete surgical staging for epithelial malignancies of the ovary, fallopian tube, primary peritoneal cancer, high-risk endometrial cancers, and for cervical cancer staging [2,3,4]. According to the NCCN (National Comprehensive Cancer Network) and Slovenian National Guidelines, the indications for para-aortic lymphadenectomy in ovarian cancer are the staging procedure in newly diagnosed invasive epithelial ovarian cancer apparently confined to an ovary or to the pelvis (apparent FIGO stage IA–IIA) or a debulking procedure in newly diagnosed invasive epithelial ovarian cancer involving the pelvis and upper abdomen (FIGO stage ≥ IIB) with suspicious or enlarged nodes, identified on preoperative CT [5,6]. 

According to the NCCN (National Comprehensive Cancer Network) and Slovenian National Guidelines, the indications for para-aortic lymphadenectomy in uterine neoplasms are newly diagnosed high-risk uterine tumor, such as deeply invasive lesions, high-grade histology, serous carcinoma, clear cell carcinoma, or carcinosarcoma. In locoregional relapse disease, isolated para-aortic lymph node recurrence is also indication for para-aortic lymph node dissection [7,8]. 

The regional status of the lymph nodes is a major prognostic factor and a decisive criterion for the use of adjuvant therapy [9]. Para-aortic lymphadenectomy as a dissection of fat-lymphatic tissue is a standard procedure performed by gynecologic oncologists or surgeons in specialized cancer centers. The mastery of para-aortic lymphadenectomy requires a certain learning curve and perfect knowledge of the usual anatomy and its possible variants. It is always necessary to consider the various pitfalls that may occur during para-aortic lymphadenectomy [10]. 

Usually, the aorta enters the abdominal cavity from the thorax through the diaphragmatic hiatus and is located to the left of the inferior vena cava. The bifurcation of the aorta and inferior vena cava is at the level of the L4-L5 vertebral bodies, where the aorta divides into the common iliac vessels, which branch further into the external and internal iliac vessels. The external iliac vessels continue below the inguinal ligament to the thigh as the vasa femoralis. The internal iliac artery and internal iliac vein divide further into their visceral branches [11]. The renal arteries arise from the aorta at the level L2 vertebrae. The right renal artery usually runs dorsally below the inferior vena cava. The renal veins usually enter into the inferior vena cava at the same level as the renal artery. The left renal vein passes in front of the aorta below the gap of the superior mesenteric artery. The inferior mesenteric artery arises from the aorta about 3–4 cm above the bifurcation of the aorta. The lumbar arteries arise in pairs from the posterior wall of the aorta. The right ovarian vein usually opens into the inferior vena cava about six to seven centimeters proximal to its root and usually one centimeter distal to the right renal veins; less commonly, it may open directly into the right renal veins. The left ovarian vein usually follows the course of the left ureter and the orifice on the left side of the aorta into the ipsilateral renal vein. The arterial ovarian trunks have a similar course, with the right one emerging from the anterior wall of the aorta about five to six centimeters above the aortic bifurcation, and the left one emerging mostly from the left renal artery [11]. The course of the vessels may be variable in the para-aortic region, and knowledge of these anatomic variants is important for an uncomplicated course of the operation. 

In para-aortic lymphadenectomy, the Cattell–Braasch maneuver is usually used to completely expose the inferior vena cava, renal veins, abdominal aorta, and inferior mesenteric artery [12]. The Cattell–Braasch maneuver begins with the transection of the lateral parietal peritoneum from the right external iliac artery to the hepatoduodenal ligament. The right ureter and right ovarian vessels must be separated from the mesocolic plane and located and marked with the vascular loop. In the next phase, the peritoneum on the right side of the duodenum is transected and the avascular surface between the inferior vena cava and the posterior side of the pancreatic head and duodenum is transected; this is followed by mobilization of the duodenum to the superior mesenteric artery and abdominal aorta. The mobilized right colon and duodenum are then placed in the physiological position, back on the right side of the abdomen for small bowel mobilization. The parietal peritoneum between the ligamentum Treitz and the right external iliac artery is visualized and transected to free the small bowel completely from the retroperitoneum. The mobilized colon and small bowel are placed on the upper abdomen and the thorax, covered with moist gauze, and then fixed with the blades of the abdominal retractor or held in place by the assistant. Special attention is needed to avoid torsion of the colon and the small bowel mesentery, as this may cause ischemia [12]. The Cattell–Braasch maneuver carries the risk of injury to the duodenum, pancreas and ascending colon, and right ureter, and could causes paralytic ileus [13]. A disadvantage of laparotomy with the Cattell–Braasch maneuver may be delayed recovery of gastrointestinal function [14], which may result in a poorer prognosis for patient discharge from the hospital.

The lymph nodes surround the aorta and inferior vena cava throughout their course and form individual nodal groups draining pelvic organs and intestinal loops. The landmarks for systematic para-aortic lymphadenectomy include, on the right side, right renal vein superiorly, mid-common iliac artery inferiorly, right ureter laterally, and aorta medially; on the left side, marks include the level of the entry of the left ovarian vein into the left renal vein superiorly, mid-common iliac artery inferiorly, left ureter laterally, and aorta medially [15]. In relation to the aorta and inferior vena cava, the lymph nodes are divided into paracaval, precaval, and retrocaval segments, which are located to the right, anterior, and posterior of the inferior vena cava, respectively; preaortic, para-aortic, and retro-aortic nodes, which are located anterior, left, and posterior to the aorta, respectively, and interaortocaval nodes, which are located between the inferior vena cava and the aorta, which in turn are divided into deep and superficial according to the course of the lumbar vessels [16]. In para-aortic lymphadenectomy, the fatty lymphatic tissue should be dissected out from preaortic, para-aortic, and retro-aortic sites, as well as from identical sites around the inferior vena cava and from the interaortocaval area [2,4,17]. 

In all patients in whom para-aortic lymphadenectomy is planned, preoperative contrast-enhanced abdominal CT should be performed, and description of possible metastatic lymph nodes and various anatomic variants must be part of the standard radiologic report. In the literature, there are very few reports on the percentage of different anatomic variants in the preoperative CT and even fewer reports on surgical series on this very important topic for a practicing surgical gynecologist [18,19]. The aim of this study was to assess the frequency of different anatomical variants and to assess how often are they described in the preoperative CT report.

## 2. Materials and Methods

From January 2021 to May 2023, 58 consecutive patients underwent para-aortic lymphadenectomy for gynecologic malignancies at the Institute of Oncology Ljubljana. In 2019, the National Committee of Gynecologic Oncology decided that we should concentrate high-risk patients requiring para-aortic lymphadenectomy in one institution. We formed a special surgical team consisting of two surgical gynecologists and one surgical oncologist who are now working together. We decided that learning curve should be at least 30 procedures, which we achieved at the end of 2020.

**Figure 1:**
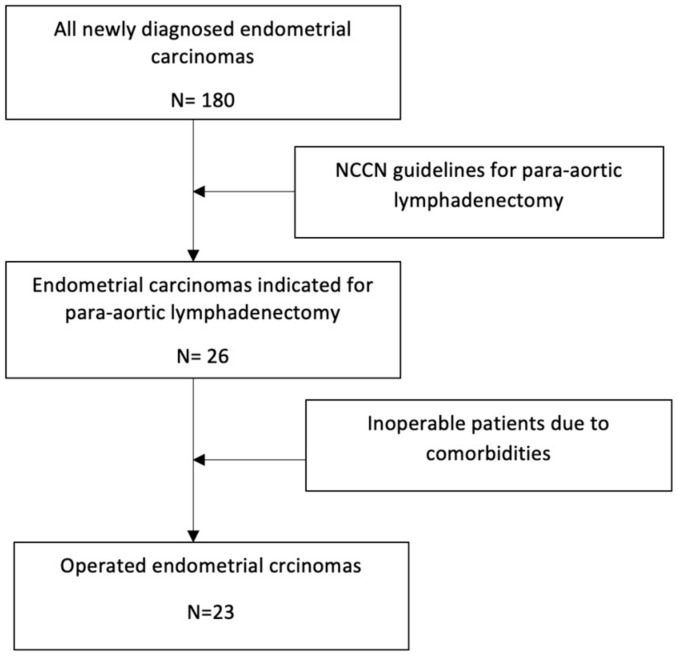
Patient selection with endometrial cancer.

**Figure 2:**
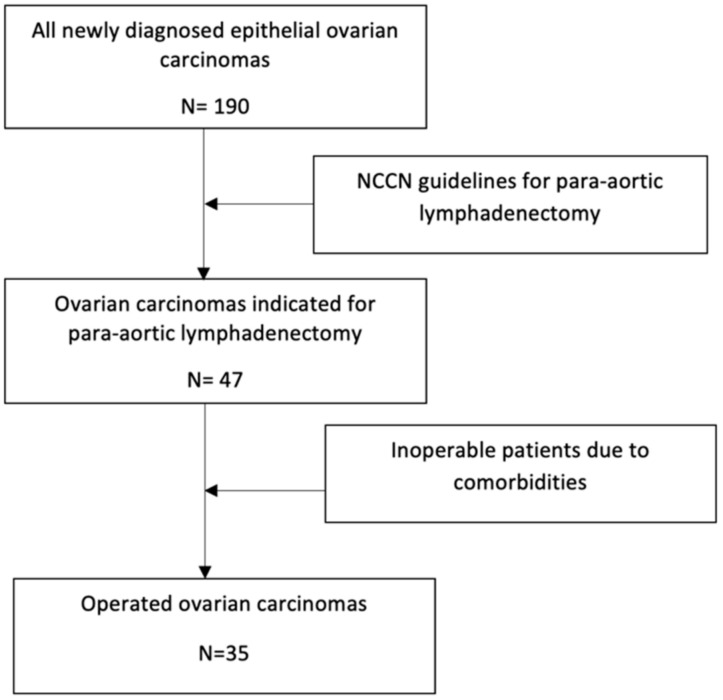
Patient selection with ovarian cancer.

We included patients with newly diagnosed ovarian and endometrial cancer (Figure 1 and Figure 2). 

For ovarian cancer, the inclusion criteria was newly diagnosed, histologically confirmed invasive epithelial ovarian cancer at CT, apparently confined to an ovary or the pelvis, FIGO stage IA to IIA [5,6].

For endometrial cancer, the inclusion criterion was a newly diagnosed high-risk uterine tumor, such as high-grade histology, serous carcinoma, clear cell carcinoma, or carcinosarcoma [7,8].

Exclusion criteria were as follows: (1) patients with recurrence of either ovarian or endometrial cancer; (2) epithelial ovarian cancer FIGO stage ≥ IIB; (3) non-epithelial ovarian cancer; histologically confirmed benign, borderline epithelial ovarian tumors and mucinous ovarian tumors; (4) endometrial carcinoma with histological grade 1 and grade 2; (5) inoperable patients who have comorbidities and, therefore, were not appropriate candidates for surgery.

**Figure 3:**
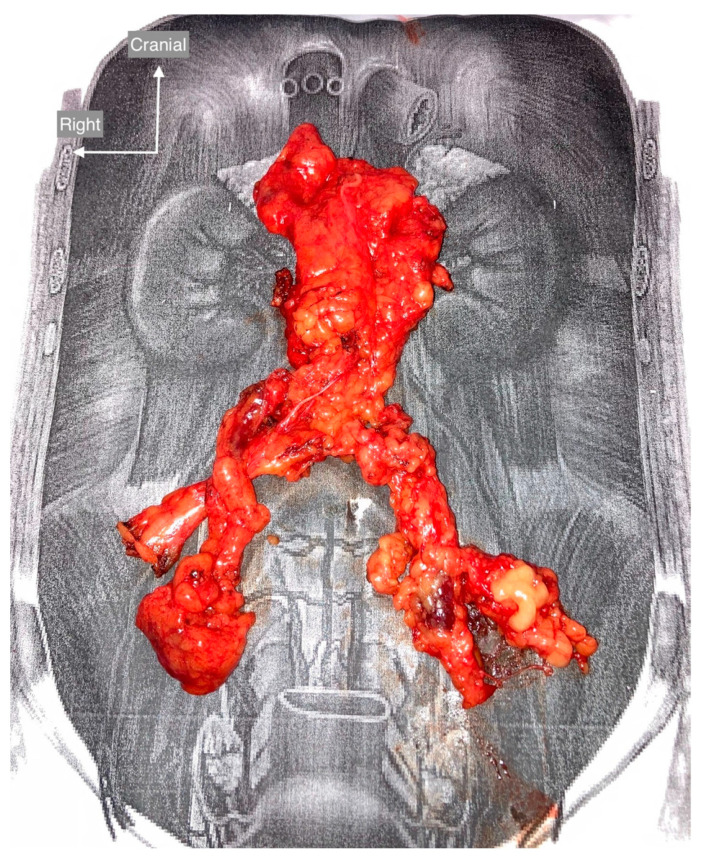
Orientation scheme with dissected tissue.

Before surgery, imaging studies were performed in all patients, including transvaginal ultrasound and contrast-enhanced CT scans of the abdomen and chest. A standard systematic surgical approach as described in [15] was performed in all patients. The dissected tissue was oriented according to a scheme and sent to pathology (Figure 3). The number of lymph nodes removed in the final histopathology report was used as a quality indicator of the adequacy of the surgical procedure. Vascular and urinary tract anatomic variants were retrospectively reviewed in the prospective institutional database, and the results were compared with the preoperative abdominal CT.

Regulatory approval for the study was obtained from the Institutional Review Board (No. ERIDNPVO-0032/2022). Signed informed consent was obtained from all patients included in the study. Patients were presented at interinstitutional tumor board meetings consisting of a gynecologic oncologist, a radiologist, a medical oncologist, a radiation oncologist, and a pathologist. The main objective of the study was to assess the correlation of different anatomical variants and their report in CT scans. Furthermore, the authors looked to note vascular and urinary tract variants that could require changes in surgical planning.

## 3. Results

Over a two-year period, 58 women underwent para-aortic lymphadenectomy for ovarian or endometrial cancer as part of a staging/therapeutic procedure. The usual clinical symptoms were nonspecific and consisted of abdominal pain, palpable mass, metrorrhagia, and fatigue due to malignancy. All patients were asymptomatic for vascular or urinary changes before surgery. Baseline patients’ characteristics are described in Table 1.

The median number of para-aortic lymph nodes removed was 31, ranging from 6 to 58. The median number of pelvic lymph nodes removed was 13, ranging from 2 to 31. Pathologic para-aortic or iliac lymph nodes were described in four patients CT, but final histopathologic examination of the lymph nodes revealed no metastases. In 9 patients, CT showed no pathologic lymph nodes, but final histopathologic examination revealed that para-aortic or iliac lymph nodes harbored metastases.

Anatomic variants in the preoperative CT were described by the radiologist in only two patients, namely a double inferior vena cava in one patient and a retro-aortic course of the left renal vein in one patient. Preoperative radiologic findings regarding pathologic lymph nodes and anatomic variants were consistent with intraoperative findings in 45 patients. Additional vascular and urinary tract variants that had not been described by a radiologist were noted in nine patients at the time of surgery. During the surgical procedure, the most surprising finding was that the right ovarian artery was seen in only two patients, whereas the left ovarian artery was not seen in any. If we disregard this, 47 (81%) patients had usual anatomy with no vascular or urinary tract variants (Figure 2).

The anatomic variants detected at para-aortic lymphadenectomy were as follows: one patient had a double inferior vena cava, four patients had accessory renal arteries, two patients were found to have a retro-aortic left renal vein, two patients had a double left ureter, one patient had a ptotic kidney in the left iliac fossa, and one patient had bilateral renal malrotation (Table 2). 

**Figure 4:**
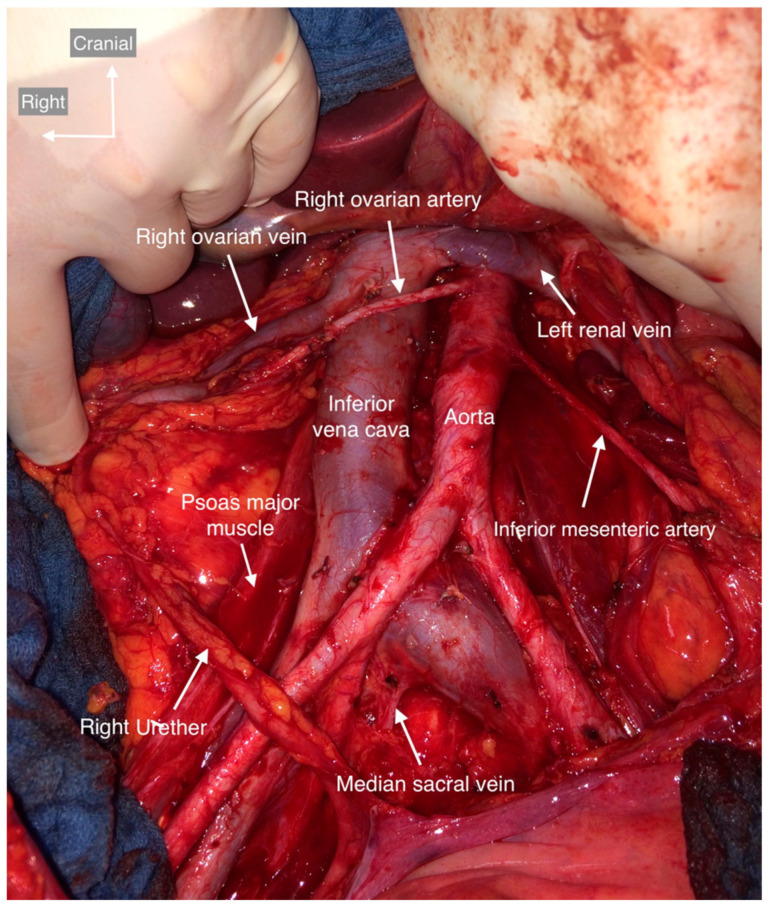
Usual vessel and urinary tract anatomy. The ventral boundary is the left renal vein; the right renal vein is not usually seen during the procedure. The right boundary is the right ureter, psoas major muscle, and ascending colon. The right ovarian vein, which arises from the inferior vena cava, and the ovarian pedicle should be separated from the right ureter and ligated at its insertion. The left border is formed by the left ureter, the psoas major muscle, and the descending colon. The inferior mesenteric artery always arises from the abdominal aorta. The dorsal border is the bifurcation of the common iliac vessels, which continues to the pelvic lymph nodes.

**Figure 5:**
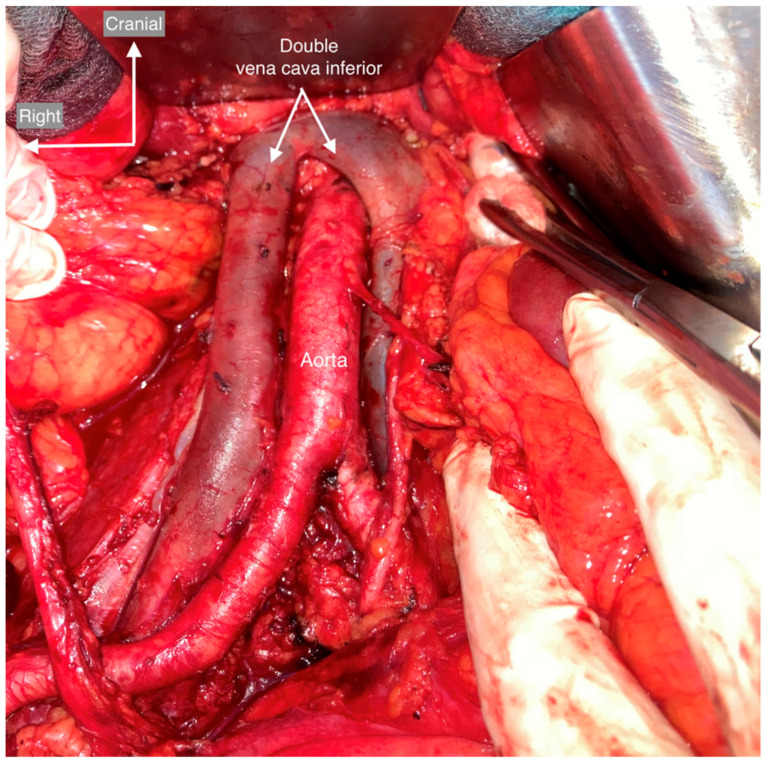
Double inferior vena cava. In a duplication of the inferior vena cava, the left vena cava crosses the aorta. This is a type 1 duplication in which the left and right trunks are symmetrical and of the same caliber. The left trunk of the inferior vena cava receives the left renal vein and continues as the preaortic trunk. The left common iliac vein is part of the duplicated inferior vena cava and joins the ascending vein as the duplicated inferior vena cava.

**Figure 6:**
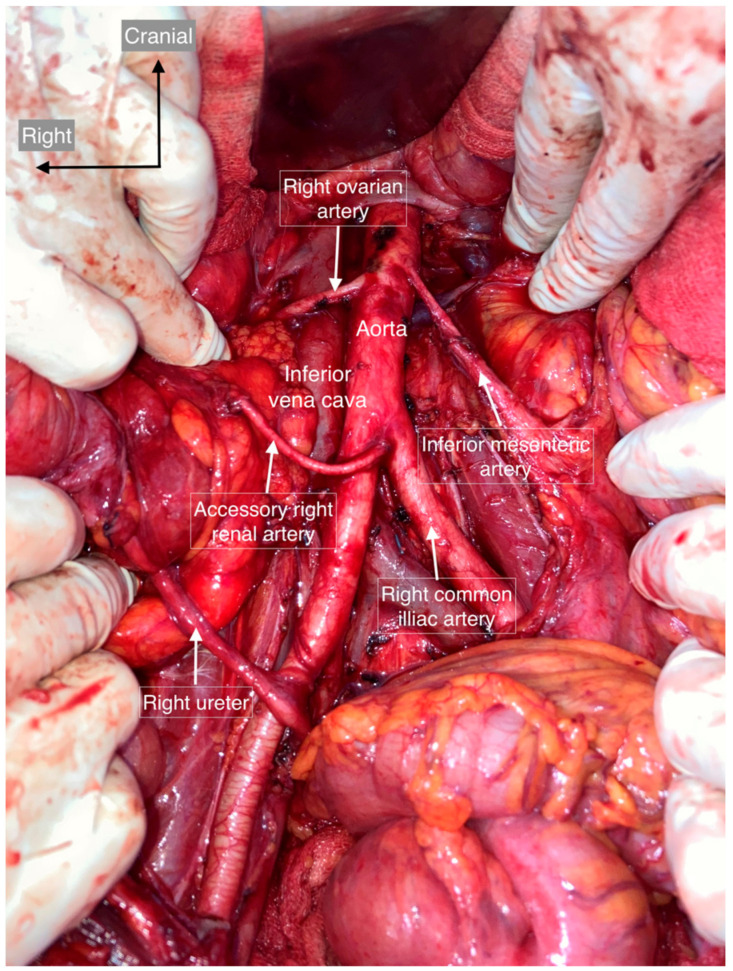
Accessory renal artery. The right accessory renal artery arises from the left common iliac artery and supplies the right superior renal hilum.

**Figure 7:**
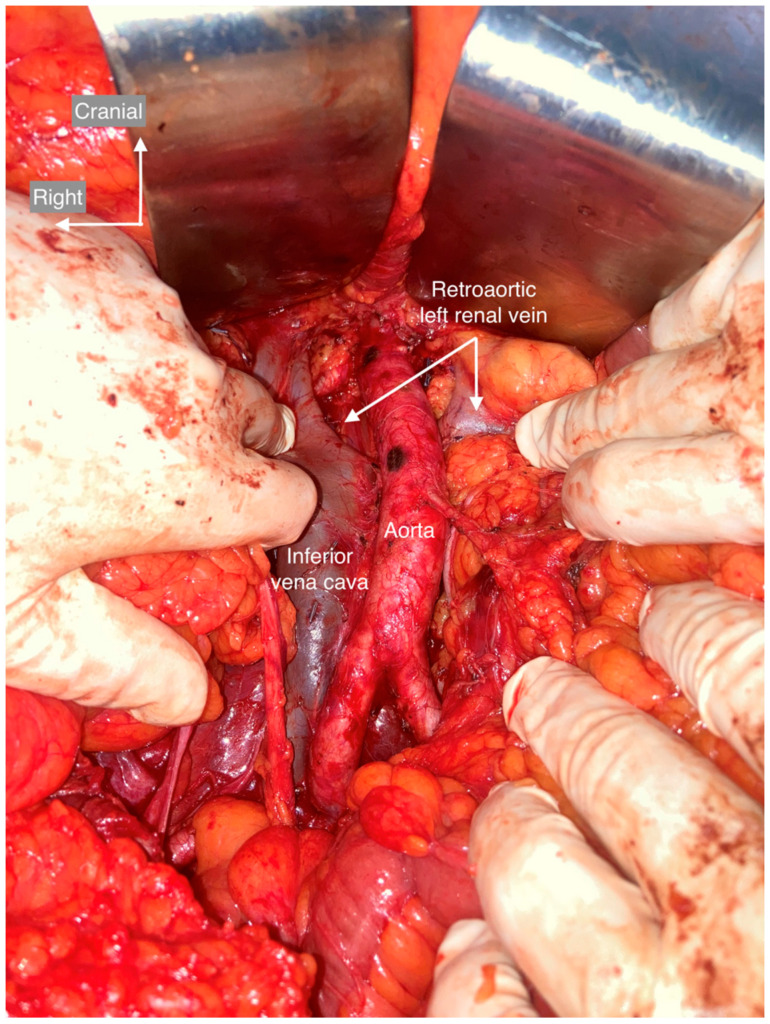
Retro-aortic left renal vein. The left renal vein has a retro-aortic course before entering inferior vena cava.

**Figure 8:**
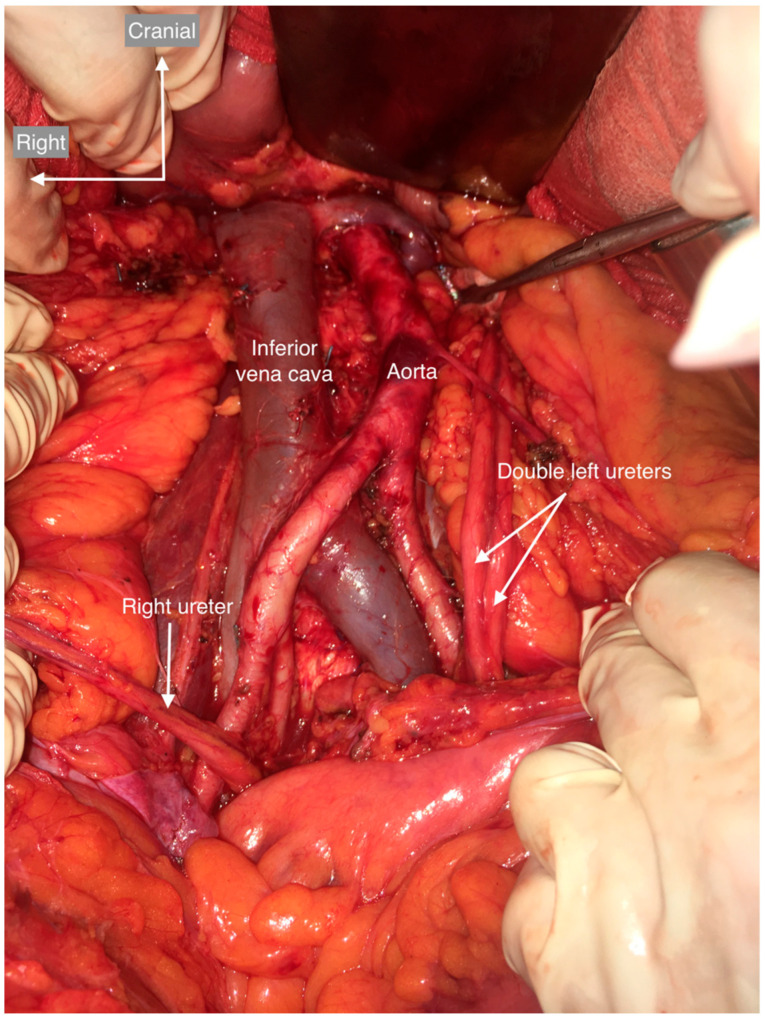
Ureter duplication. Complete duplication of the left ureter. None of the ureters were dilated, indicating that there was no vesicoureteral reflux or ectopic ureterocele.

**Figure 9:**
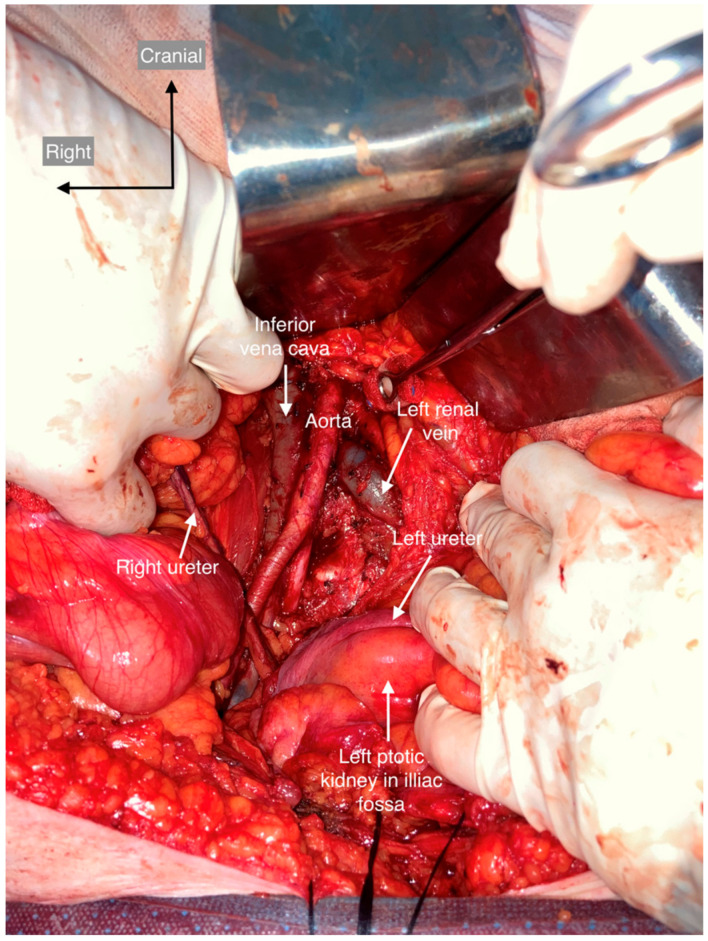
Ptotic kidney in the iliac fossa. The position of the left kidney was lower than normal and located in the left iliac fossa. The medial end of the left kidney was anterior to the sacral promontory, as low as the bifurcation of the common iliac artery. The left ureter was located more anteriorly, medially, and lower than normal as it descended into the bladder. The arterial vasculature of the ptotic kidney was not altered. The left renal vein had a retro-aortic course. The right kidney was in normal position and rotation.

**Figure 10:**
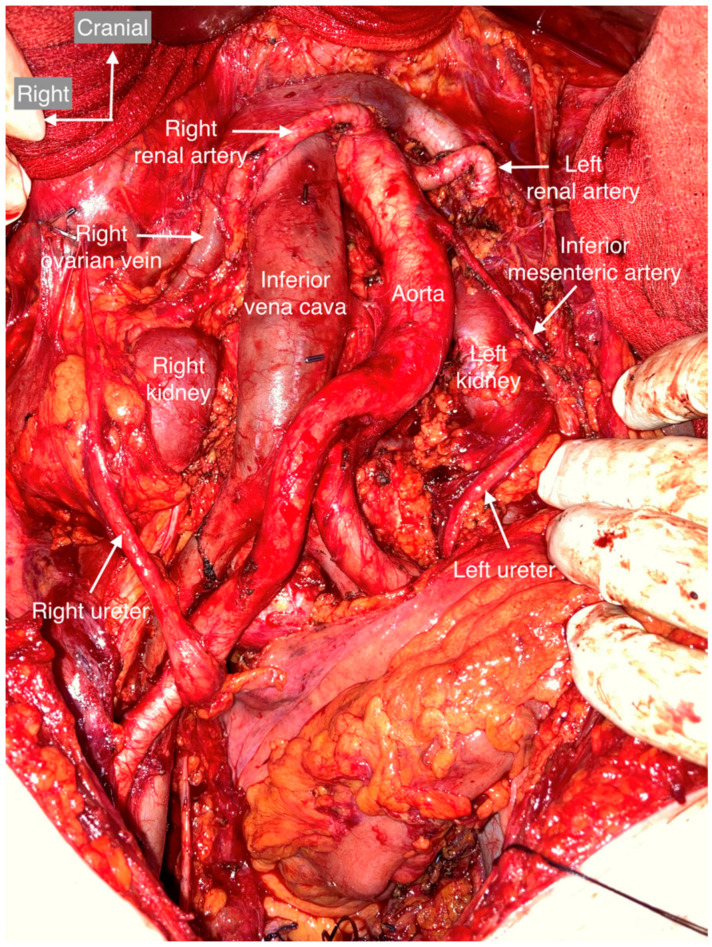
Bilateral kidney malrotation. An anomalous kidney rotated round its vertical axis caused the hilus to be directed posterolaterally rather than anteromedially. The position of the malrotated kidneys was normal at the level of the L2-L3 vertebrae.

All anatomic variants are shown and described in the following figures (Figure 4, Figure 5, Figure 6, Figure 7, Figure 8, Figure 9 and Figure 10). None of the patients had genital anomalies. 

## 4. Discussion

The extent of tumor burden and patient’s clinical status could impair the feasibility of conducting an extensive surgery safely, especially in elderly, malnourished patients with comorbidities. Patients with reduced physiologic reserve associated with frailty and comorbidities are at higher risk for significant intraoperative and postoperative complications. In ovarian cancers where frail patients are unable to achieve complete cytoreduction, it is prudent to consider the use of neoadjuvant chemotherapy [21].

Vascular and urinary tract variants are important because they can affect the surgical plan and lead to incidental intraoperative and postoperative complications. The vast majority of studies describing anatomic variants of the abdominal para-aortic region have been performed on cadavers [20,22,23,24,25]. There are very few studies of anatomic variants of this region performed with contrast-enhanced abdominal CT, which is a routine preoperative staging method [18,26,27], and even fewer studies of the frequency of surgically detected anatomic variants during para-aortic lymphadenectomy [17]. Our study compares a routine interpretation of abdominal CT by radiologists and correlation with intraoperatively detected vascular and urinary tract variants. 

In our study of 58 patients, anatomic variants were described in the preoperative CT by a radiologist in only two patients, whereas, during surgery, 18.9% (11/58) vascular and urinary tract variants were found, with 12.1% (7/58) in vascular anatomy and 6.8% (4/58) in urinary tract anatomy. Aljabri reported that the prevalence of significant venous and arterial anomalies associated with the abdominal aorta, inferior vena cava, and iliac arteries detected by contrast-enhanced CT was 5.65%. He concluded that contrast-enhanced CT is a reliable method for accurate identification of these vascular anomalies because the frequency is consistent with that found in autopsies [18]. If we exclude the four patients with accessory renal arteries, the prevalence of vascular anatomy variants in our series is 5.2%, which is consistent with the data published by Aljabri et al [18]. 

In our series of patients, one had a duplication of the inferior vena cava, which was also described by radiologists on preoperative abdominal CT. The two most common congenital anomalies in the inferior vena cava system are transposition and duplication of the inferior vena cava. Duplication of the inferior vena cava, with a prevalence of 0.7% according to the literature, is an extremely rare anatomic variant that has significant implications for surgical oncology [28].

In two of our patients, the left renal vein was positioned retro-aortically, so that the upper border of the lymph node dissection was not clearly visible as usual. In one patient, this variant was also routinely described by radiologists at CT. The prevalence of retro-aortic left renal veins is 2.1% in the literature [29]. When the left renal vein is retro-aortic, the upper ventral border of para-aortic lymph node dissection may be missed, and the superior mesenteric artery or left renal artery may be injured during dissection [30].

The most common vascular variant in our study was the presence of an accessory renal artery, which was found in 4 patients (6.8%), but none of them was routinely described by the radiologist on the CT scan. Accessory renal arteries are a common variant of the renal artery and occur unilaterally in 20–30% of the general population and bilaterally in 10% of the population [31]. They may be unilateral or bilateral and there may be varying numbers of one to several of them in an individual [32]. At the dorsal margin of the para-aortic lymphadenectomy, the surgeon should look carefully for accessory renal arteries, which may originate from the aorta or the common iliac artery [33]. Accessory renal arteries may also be associated with ovarian vascular variants. Accessory renal arteries may enter the upper or lower poles of the kidneys. When they enter the lower pole, they may obstruct the ureter, resulting in hydronephrosis [34].

Abnormalities of the kidneys and urinary tract occur in 3.3% to 11.1% of the population [35]. In our study, we noted four patients (6.8%) with urinary tract variants, and none of them were routinely described by the abdominal CT. CT urography has emerged as the preferred imaging modality for evaluation of the retroperitoneal organs, particularly the kidney, and has proven invaluable in the diagnosis of urinary tract abnormalities. Abdominal CT with or without contrast in not specifically tailored for comprehensive assessment of the upper and lower urinary tract [36,37]. During embryonic development, the kidneys ascend and rotate anteromedially. If malrotation occurs, there is an abnormal position of the kidney, which can be unilateral or bilateral [38]. A common ectopia in the renal position is the pelvic kidney, which occurs in 1/3000 autopsies. On clinical examination, it may be mistaken for a pelvic tumor and removed unnecessarily. During surgical procedures, the pelvic kidney presents a challenge because of the greater risk of injuring aberrant vessels, nerves, or visceral tissues [39]. A ptotic kidney may go unnoticed during life and be an incidental finding during surgery or imaging, like it was in one of our patients.

Rotational variants are a rare occurrence. They play an important role in surgical planning, as the vessels and ureters are positioned differently, and possible iatrogenic trauma can occur during the surgical procedure. In our study, we observed a patient with bilateral posterolateral renal malrotation. At the fourth week of gestation, the kidneys are close together and the hilum is anterior in the pelvis. At the ninth week of gestation, there is an ascent and a 90-degree rotation occurs, and the kidneys are spaced apart with the hilum directed anteromedially [38]. When malrotation occurs, variants are classified as nonrotation, incomplete rotation, reverse and transverse rotation, or over-rotation, with nonrotation and incomplete rotation being the most common [40].

Ureteral duplication is the most common congenital urinary tract anomaly, which can be complete or incomplete [41]. In our study, we incidentally found a double ureter intraoperatively in two patients. Incomplete duplication is three times more common than complete duplication, with a prevalence of 0.8% [41]. Clinical complications of complete ureteral duplication include ectopic ureterocele, vesicoureteral reflux, and ectopic ureteral insertion [42].

A limitation of our study is the relatively small number of patients. Nevertheless, the frequency of obtained anatomical variants is consistent with the data in the literature [28,29,31,35,41]. We have deliberately not addressed the issue of anatomical variants in the nervous system of this specific anatomical region, since from a surgical point of view this is of less importance in female patients than in male patients, in whom injuries to the autonomic nervous system lead to considerable morbidity (erectile and ejaculatory dysfunction) and reduced quality of life. On the other hand, the strength of the study is the experience of a single tertiary center with the same dedicated surgical team and approach.

One perspective in complex surgical procedures could be artificial intelligence (AI) and AI-assisted surgery. AI involves the study of algorithms that provide machines with problem-solving abilities, the ability to recognize visual elements in images, and the ability to make predictions based on statistical inference. In medicine, AI is demonstrating its capabilities by analyzing large data sets from patient records, radiology scans, or surgical videos, and using this information for the purpose of recognition, classification, and prediction [43]. In addition, AI could play a pivotal role in improving surgical decision-making processes, both pre- and postoperativelys, as well as during the surgical procedure itself [44]. Through features, such as tool highlighting, surgery monitoring, and alert generation, AI-assisted surgical systems will be able to find a path for each patient’s surgical needs and facilitate and streamline surgical procedures. In surgical fields, such as laparoscopy and robotic surgery, AI will prosper because it will be able to provide real-time guidance and information via a video screen during the procedure [45]. AI could help surgeons in risk stratification for complex surgeries, such as para-aortic lymphadenectomy, and assist them in predicting anatomic and vascular variations. It is important to emphasize that AI-assisted surgery is not intended to replace the surgeon, but rather to serve as a valuable assistant in the surgical procedure.

## 5. Conclusions

Vascular and urinary tract anatomic variants are common and may be found in one out of five patients during para-aortic lymphadenectomy, so the surgeon should always consider them. The most common vascular variant is the accessory renal artery, which is usually not detected on preoperative imaging. An abdominal CT should be interpreted jointly by a radiologist and a surgical gynecologist whenever possible. For optimal intraoperative management, it is strongly recommended that precise dissection be performed to facilitate exposure and provide valuable insight for potential vascular repair.

## Figures and Tables

**Table 1:** Overview of patients baseline characteristics.

Characteristics	Number/Median Values (IQR)
Total number of patients	58
Endometrial cancer	23
Ovarian cancer	35
Age in years	65 (21–83) (5–72)
Body mass index	25.8 (19.3–41.3) (22.1–29.8)
Removed para-aortic lymph nodes	31 (6–58) (28–37)
Removed pelvic lymph nodes	13 (2–31) (10–19)

**Table 2:** Anatomic variants detected at para-aortic lymphadenectomy.

Anatomic Variants		Our Results	Literature
Double inferior vena cava		1/58 (1.7%)	0.7% [15]
Retro-aortic left renal vein		2/58 (3.4%)	2.1% [20]
Accessory renal arteries		4/58 (6.8%)	20–30% [16]
Kidneys and urinary tract	Double left ureter	2/58 (3.4%)	3.3–11.1% [19]
	Ptotic kidney	1/58 (1.7%)	
	Renal malrotation	1/58 (1.7%)	

## Data Availability

The data presented in this study are available on request from the corresponding author. The data are not publicly available due to the privacy of the patients.
